# Preliminary report of directly observed treatment, short course in tuberculous meningitis

**DOI:** 10.4103/0972-2327.61279

**Published:** 2010

**Authors:** Thomas Iype, Sinchu Chacko, Sivadasan Raghavan, Robert Mathew, Madhusudanan Mohan

**Affiliations:** Department of Neurology, Medical College, Thiruvananthapuram, India

**Keywords:** Cerebrospinal fluid, diagnosis, imaging, treatment, tuberculous meningitis

## Abstract

**Background:**

Diagnosis of tuberculous meningitis (TBM) is a challenge because of the manifold clinical presentation, and diagnosis is often delayed.

**Objectives:**

We wanted to share our experience of directly observed treatment short course (DOTS) in TBM. We did a retrospective analysis to look at the presentation, management and outcome of TBM patients from November 2006 to April 2008.

**Materials and Methods:**

TBM was diagnosed based on clinical criteria. We excluded patients with HIV.

**Results:**

We had 11 patients on DOTS regime. One died following hepatitis and another patient died of unrelated gastroenteritis. The only patient on daily regime died. Our patients generally presented late, at a median duration 20 days from onset of symptoms, and 50% had stage 3 disease at presentation. The median delay in diagnosis was 4.5 days.

**Discussion:**

We found DOTS to be effective in TBM but not without side effects.

## Introduction

Tuberculous meningitis (TBM) is a common neurological condition causing high mortality and morbidity in developing countries. We wish to share our experience of directly observed treatment, short course (DOTS), in TBM.

Diagnosis of TBM is a challenge because of the manifold clinical presentation and the poor sensitivity and specificity of the various diagnostic tests. Even with the best techniques acid-fast bacillus (AFB) stain was positive in only 58% and culture for Mycobacterium tuberculosis in 71% of instances.[1] This can result in delayed diagnosis and, therefore, increase in morbidity and mortality. We also looked at the varied clinical presentations of TBM.

## Materials and Methods

This is a retrospective study of patients with TBM diagnosed in our department. The study period was from November 2006 to April 2008. We made a diagnosis of TBM when patients with meningitis (CSF pleocytosis) presented to us with symptoms lasting 7 days or more, with negative Gram's stain and India ink stains, and sterile bacterial and fungal cultures, plus one or more of the following: cranial CT scan consistent with TBM (hydrocephalus, basal meningeal enhancement, ring-enhancing lesion), chest radiograph consistent with active pulmonary tuberculosis, and good response to antituberculous chemotherapy. The duration of symptoms was calculated as the period from onset of the first symptom to presentation to our center. All our patients satisfied the clinical criteria for TBM.[2] We staged patients according to the Medical Research Council staging system.[3]

The department policy was to give the category 1 DOTS regimen[4] for 9 months for all patients with TBM, along with corticosteroids for the first month. We excluded HIV-positive patients. In doubtful cases, where bacterial meningitis was a possibility, we gave intravenous ceftriaxone 2 g twice daily and watched for clinical response; if needed, we did a repeat CSF study. In bacterial meningitis, the earliest response to intravenous antibiotics is an increase in CSF glucose, which is seen within 48 h of institution of treatment.

## Results

We had 12 patients of TBM during this period. The male:female ratio was 7:5. The median age of the patients was 28.5 years [Table 1]. Patients had symptoms for a median period of 20 days prior to presentation [Table 1]. Fifty percent of our patients had stage 3 disease [Table 1]. Three of our patients had extracranial tuberculosis: one (case no. 6) had caries spine, one (case no. 7) had pulmonary tuberculosis, and one (case no. 10) had histopathologically proven caseous granulomatous axillary lymphadenitis.

**Table 1:** Demography and imaging

Sex	Age	Diagnosis	Dur. of PC	MRC Stage	Delay in diagnosis (days)	CT scan	MRI scan
F	35	TBM	60	1	2	WNL	ND
F	44	TBM with optochiasmatic arachnoiditis	60	1	1	WNL	Plain scan WNL
M	45	TBM with hydrocephalus	10	1	0	WNL	Leptomeningeal enhancement; dilatation of lateral and third ventricle
M	27	TBM with endarteritis and hydrocephalus	19	3	12	Hydrocephalus, basal meningeal enhancement; multiple lacunar infarcts in the deep grey matter	ND
M	13	TBM	8	1	5	WNL	ND
F	29	TBM with arachnoiditis, caries spine	120	3	1	ND	Dilatation of ventricles with periventricular symmetric hyperintense shadows
M	28	TBM with hydrocephalus, PT	10	3	7	Hydrocephalus	Dilatation of lateral and III ventrilcles with normal 4th ventricle.
F	23	TBM with optochiasmatic arachnoiditis	10	3	4	WNL	Multiple ring-enhancing lesions
M	48	TBM with multiple granuloma	60	2	5	Basal meningeal enhancement	Multiple ring- enhancing lesions
F	27	TBM	21	2	13	WNL	ND
M	40	TBM with conglomerate granuloma with PT and caseating lymph node granuloma	25	3	1	Infarct in the external capsule on right side, posterior limb of internal capsule on left	Basal meningeal enhancement, conglomerate small enhancing lesion in right sylvian fissure
M	19	TBM	7	3	24	WNL	WNL

Dur-duration; PC-presenting complaints; MRC-Medical Research Council; PT-pulmonary tuberculosis; WNL-within normal limits; ND-not done.

There was a median delay of 4.5 days from the initial CSF study to initiation of antituberculous treatment (ATT) [Table 1]. Three patients died. One patient (case no. 4) died in the hospital due to drug-induced hepatitis 6 days after DOTS had been started. The second patient (case no. 6) had actually started improving with DOTS, but died due to an unrelated cause (gastroenteritis and aspiration pneumonia). The third patient (case no. 9), who presented to us 60 days after the onset of illness, had multiple granulomas on MRI and was very sick. He received daily ATT rather than DOTS right from the beginning. He however died 6 days after start of treatment. All except this patient (case no. 9) were given the DOTS regimen. The patients who died had severe stages of disease [Table 1]. The other nine patients had a mean follow-up of 361 days (range: 212–731 days) on DOTS. Two patients had bilateral optic atrophy, which produced mild visual impairment but did not compromise their activities of daily living. None of the others had any functional disability.

Two of our patients [Table 2] had polymorph-dominant (i.e., polymorphs >50%) pleocytosis; the duration of illness in both cases was ≤10 days. Two patients had CSF glucose between 10 and 20 mg%. We had CSF adenosine deaminase (ADA) levels estimated in four patients and all showed values ≥10 U/l [Table 2]. Polymerase chain reaction (PCR) for tuberculosis Polymerase chain reaction (PCR) for tuberculosis was done on three samples and was negative in all [Table 2]. None of our patients had a positive microscopy for acid-fast bacilli or positive mycobacterial culture.

**Table 2:** CSF results

TC 1	DC P 1	Prot 1	Sug 1	ADA 1	TB PCR	TC 2	DC P 2	Prot 2	Sug 2	IV ceftriaxone 2 g twice daily	Days of R_X_
560	2	70	33	ND	ND	ND	ND	NA	NA	No	NA
130	3	72	56	ND	ND	ND	ND	NA	NA	No	NA
220	2	95	23	ND	ND	1030	2	102	37	Yes	*6
60	2	97	30	21.3	Neg	480	38	71	29	Yes	†7
360	85	78	60	ND	ND	ND	ND	NA	NA	Yes	NA
20	50	208	43	ND	ND	ND	ND	NA	NA	No	NA
680	8	353	76	ND	ND	ND	ND	NA	NA	No	NA
50	97	47	11	ND	ND	160	44	174	0	No	*10
140	12	25	29	10	ND	250	70	132	23	Yes	†4
180	5	52	52	ND	ND	ND	ND	NA	NA	Yes	NA
90	10	210	15	15.5	Neg	100	40	123	22	No	*20
130	21	60	24	10.6	Neg	300	95	35	27	No	*15

TC 1-Total count of first CSF study; DC P 1-percentage of polymorphs in first CSF study; Prot 1- protein of first CSF study; Sug 1-sugar of first CSF study; TC 2-total count of second CSF study; DC P 2-percentage of polymorphs in second CSF study; Prot 2-protein of second CSF study; Sug 2-sugar of second CSF study; Days of Rx-days of treatment prior to repeat CSF study

(* indicates that CSF study followed DOTS

† indicates that it followed IV ceftriaxone 2 g twice daily on)

NA-not applicable.

One patient (patient no. 3) was given a combination of intravenous (IV) ceftriaxone and DOTS for 7 days. Five patients, suspected to have partially treated bacterial meningitis, were put on antipyogenic regimen (IV ceftriaxone 2 g twice daily) [Table 2] at the time of admission to our department. Four of them had lymphocyte-dominant pleocytosis [Table 2]. ATT was started subsequently as there was inadequate clinical response. We initiated DOTS in five patients and daily ATT in one patient from the very beginning.

We repeated the CSF study in two cases (patient nos. 4 and 9); these two patients were on IV ceftriaxone but did not show any response. In both, the CSF glucose decreased although the patients had been on antibiotics for 4 days; this was interpreted as unresponsiveness to the antipyogenic regimen [Table 2]. We repeated the CSF examination in four patients (patient nos. 3, 8, 11, and 12) after initiating DOTS. The CSF cell count increased by 3–5 times in all of these patients after a mean interval of 12.75 days (range 6–20) [Table 2]. The CSF protein decreased in two patients and increased in the other two.

Eleven of our 12 patients had CT brain done at admission. Four patients out of the 11 (36.36%) showed abnormalities [Table 1]. MRI was performed late in 8 out of the 12 patients when there was some diagnostic confusion. It was abnormal in six out of the eight patients (75%) [Table 1]. Seven patients had MRI as well as CT scan. MRI demonstrated leptomeningeal enhancement in one case (patient no. 3), while CT scan did not [Table 1]. Similarly, in three patients (patient nos. 8, 9, and 11), MRI was able to identify additional granulomata that were not evident on CT scan [Table 1].

## Discussion

One patient on DOTS had an adverse drug reaction in the form of fatal hepatitis. We found DOTS to be effective in TBM (90.9%). We had good follow-up through the RNTCP network and there were no dropouts from our sample. There was very little morbidity even though most of our patients had advanced disease.

In our series, there was a high prevalence of extracranial tuberculosis, which gave a clue to the diagnosis. This emphasizes the importance of a thorough search for tuberculosis elsewhere in patients with suspected TBM. As in other case series, some TBM patients in our study had polymorph-dominant pleocytosis; this was especially seen in patients presenting in the first 10 days of illness.[5] TB PCR had a low yield in our series. PCR sensitivity varies from 56% to 90% and the specificity from 88% to 100%.[6] In some laboratories, the sensitivity of CSF TB PCR may not exceed 50%.[7]

Using a combination of DOTS and oral prednisolone we found a trend toward a marked increase in pleocytosis, which reached a maximum at the end of the first week. Similarly, the CSF protein was high at the end of first week after starting antituberculous drugs and steroids and remained high during the middle of second week. This suggests that CSF study should be repeated in patients with suspected TBM after 2 weeks of treatment with DOTS and steroids to look for a decrease in protein and an increase in the CSF glucose/blood glucose ratio, which would be suggestive of response. This recommendation needs verification in a larger prospective study. Some patients showed a paradoxical increase in the percentage of polymorphs, which is thought to be a response to ATT.[8]

The high sensitivity of CSF ADA test that was seen in this study is consistent with the available literature showing that ADA can have sensitivity and specificity as high as 83% and 95%, respectively.[9] Moreover it is relatively inexpensive and more readily available than PCR. Contrast-enhanced MRI is more likely than contrast-enhanced CT scan to reveal abnormalities in TBM. Hence, MRI with contrast should be part of the workup of suspected cases of TBM.

CSF glucose less than 20 mg/100 ml is rare in TBM[10] and hence this finding should suggest bacterial meningitis. Two of our patients who had low CSF glucose had ring-enhancing lesions on neuroimaging, which is suggestive of tuberculous etiology [Table 1].

Most of our patients presented to us late after a prolonged course of illness and the majority had stage 3 disease. None of these patients had been diagnosed as TBM even though all of them had sought medical help elsewhere.

In conclusion, DOTS is effective in patients with TBM. Although one of our patients had fatal hepatitis, the side effects of DOTS was generally low. MRI scan with contrast study is an invaluable tool in the diagnosis of TBM and can pick up findings that can be missed in a CT scan. CSF ADA estimation in the CSF is more rewarding than CSF PCR. A transient elevation of cell count and protein in the CSF in the initial 1–2 weeks is consistent with the diagnosis of TBM and should not make one think of alternative diagnoses. Looking for evidence of extracranial tuberculosis is very fruitful, especially when the conventional tests for diagnosing TBM are negative.
